# Diffusion Model-based Medical Image Generation as a Potential Data Augmentation Strategy for AI Applications

**DOI:** 10.2174/0115734056401610250827114351

**Published:** 2025-09-01

**Authors:** Zijian Cao, Jueye Zhang, Chen Lin, Tian Li, Hao Wu, Yibao Zhang

**Affiliations:** 1 Institute of Medical Technology, Peking University Health Science Center, Beijing 100191, China; 2 State Key Laboratory of Nuclear Physics and Technology, Peking University School of Physics, Beijing 100871, China; 3 Department of Health Technology and Informatics, The Hong Kong Polytechnic University, Hong Kong SAR, 999077, China; 4 Key Laboratory of Carcinogenesis and Translational Research (Ministry of Education/Beijing), Department of Radiation Oncology, Peking University Cancer Hospital & Institute, Beijing 100142, China

**Keywords:** Image generation, Diffusion models, Medical radiology, Data augmentation, Artificial intelligence, AI training

## Abstract

**Introduction:**

This study explored a generative image synthesis method based on diffusion models, potentially providing a low-cost and high-efficiency training data augmentation strategy for medical artificial intelligence (AI) applications.

**Methods:**

The MedMNIST v2 dataset was utilized as a small-volume training dataset under low-performance computing conditions. Based on the characteristics of existing samples, new medical images were synthesized using the proposed annotated diffusion model. In addition to observational assessment, quantitative evaluation was performed based on the gradient descent of the loss function during the generation process and the Fréchet Inception Distance (FID), using various loss functions and feature vector dimensions.

**Results:**

Compared to the original data, the proposed diffusion model successfully generated medical images of similar styles but with dramatically varied anatomic details. The model trained with the Huber loss function achieved a higher FID of 15.2 at a feature vector dimension of 2048, compared with the model trained with the L2 loss function, which achieved the best FID of 0.85 at a feature vector dimension of 64.

**Discussion:**

The use of the Huber loss enhanced model robustness, while FID values indicated acceptable similarity between generated and real images. Future work should explore the application of these models to more complex datasets and clinical scenarios.

**Conclusion:**

This study demonstrated that diffusion model-based medical image synthesis is potentially applicable as an augmentation strategy for AI, particularly in situations where access to real clinical data is limited. Optimal training parameters were also proposed by evaluating the dimensionality of feature vectors in FID calculations and the complexity of loss functions.

## 1 INTRODUCTION

Artificial intelligence (AI) has been gradually applied to various medical scenarios, such as computer-aided diagnosis and surgical navigation [1], as well as structure delineation and treatment planning for radiotherapy [2]. The training, vali-dation, and testing of AI models rely heavily on the amount and quality of medical data, including images. However, the accumulation of clinical data can be time-consuming, finan-cially costly, ethnically contradictory, and administratively risky [3], which prevents the effective application of AI, especially for rare diseases [4], emerging infectious diseases [5], and special populations, such as children [6].

The traditional generative models, such as Generative Adversarial Networks [7-9] (GANs), Variational Auto-encoders [10] (VAEs), and flow models [11], have provided potential solutions to the aforementioned challenges. These models can generate new samples by approximating the distribution of the original data. For instance, GANs employ a generator and a discriminator that engage in a competitive learning process to improve the quality of generated data. VAEs utilize variational inference and an encoder-decoder structure to optimize the variational lower bound and generate images. Flow models are built upon VAEs by learning and optimizing data transformation functions to generate images. As a relatively new approach gradually introduced to medical scenarios, the diffusion model was initially developed to describe the diffusion process of liquid molecules in space [12]. By incorporating regularization principles, diffusion models inject noise into the original data and reconstruct the noise-free data using noisy samples, based on an autoencoder framework. Theoretically, by capturing the complex features in medical images, diffusion models can generate an unlimited number of new images with high visual similarity and detailed diversity compared to real images. Therefore, the diffusion models potentially provide new approaches for more efficient data augmentation in medical image-based AI applications, which have not been well investigated. Therefore, this work aims to investigate the feasibility and effectiveness of medical image synthesis based on diffusion models.

## 2 MATERIALS AND METHODS

### 2.1 Study Design

Based on publicly available medical image datasets and computer simulations, this study is an experimental work that is exempt from institutional review and informed consent, as it utilizes the diffusion method.

### 2.2 Medical Image Dataset

This study used the ChestMNIST dataset from MedMNIST v2 [13], which included 78,468 chest X-ray images. All images were preprocessed to a small size of 28×28 (2D). The original visual features were retained, and the primary data formats were covered. The visual recognizability was preserved for preliminary assessment and evaluation of image quality. Considering the potential latent space inherent in the dataset, including variabilities such as setup positions, body mass index (BMI), and health status, neither artificial intervention nor additional processing was performed on the samples to facilitate the potential contribution of these special samples to the correction and adjustment of the models.

### 2.3 Annotated Diffusion Model

The annotated diffusion model was used as the algorithm framework, the principle of which can be divided into two processes: forward learning and backward propagation based on DDPM [12].

During the forward learning process, the model randomly sampled a data point x_0_ from the actually unknown and potentially complex data distribution q(x_0_). A noise level t (*i.e*., a random time step) was uniformly sampled between time steps 1 and T. The noise was sampled from a Gaussian distribution and was used to corrupt the input at level t. A neural network was then employed to predict this noise based on the corrupted image x_t_ (*i.e*., the noise applied to x_0_ according to the known schedule β_t_).

The loss function is defined by Eq. (**1**):

**Table:** 

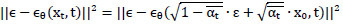	(1)

The above process was iterated until the model converged.

After training, the generative model performed backward propagation from Gaussian noise, attempting to iteratively sample and generate medical images. For a given Gaussian noise z, it was treated as the corrupted image x_t_, which was assumed to be the result obtained through the aforementioned forward training process. Within the range of steps from 1 to T, the model predicted the preceding image x_t-1_ based on the known image x_t_.

The updated formula (2) for x_t-1_, in the context of a diffusion model, during the reverse process can be expressed as follows:

**Table:** 

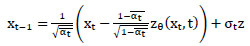	(2)

The above process was iterated until the original image x_ 0_ was ultimately reconstructed.

This study employed a U-Net architecture for the neural network model, where Residual Network (ResNet) blocks were used as the fundamental building blocks of both the encoder and decoder. The feature maps obtained from the encoder were upsampled and integrated through transposed convolutional layers and skip connections. The attention module incorporated a multi-head attention mechanism based on the transformer architecture.

The process of network construction included applying convolutional layers to a batch of noisy images and computing the positional embeddings corresponding to the noise levels. A series of downsampling stages was then applied. Each downsampling stage consisted of two ResNet blocks, a group normalization, an attention module, residual connections, and a downsampling operation. In the middle of the network, the ResNet blocks were applied again, interleaved with attention modules. A series of upsampling stages was then applied. Each upsampling stage was composed of two ResNet blocks, a group normalization, an attention module, residual connections, and an upsampling operation. Finally, the ResNet blocks and the convolutional layers were applied to produce the output.

Fig. (**1**) illustrates the learning principles and the iterative processes of the proposed diffusion model:

By gradually adding random noise to the original sample x_0_, the final Gaussian noise *z* became completely meaningless. Then the diffusion model generated a new image by denoising gradually from the Gaussian noise

### 2.4 Parameter Selection and Evaluation Metrics

In this study, the proportion of noise and original samples in the mixed samples was controlled by adjusting the β value during each round of model training. This parameter was used to determine the intensity of the noise added in each diffusion process, and its value was set from 0.0001 to 0.02. The Huber loss function was primarily used for model training. The experiments were conducted on a local NVIDIA GeForce RTX 2060 Graphics Processing Unit (GPU). The ChestMNIST dataset was divided into 2,453 batches, each containing a batch size of last drop. The loss function was trained for 40 epochs, with each epoch consisting of 300 steps. The experimental results were quantitatively analyzed using the loss function and the Fréchet Inception Distance (FID). As a non-negative metric, FID was used to measure the similarity between the generated images and the real images. The higher quality of the generated images can be indicated by a lower FID value, implying greater similarity to the original images. An FID value of zero indi-cates the original sample. As complementary to the quantitative assessment, three experienced clinicians (a radiation onco-logist, a therapist, and a radiographer) were invited to visually evaluate the AI-generated images qualitatively.

## 3 RESULTS

### 3.1 Visual Display of Training Results

Using the aforementioned settings, each model training session took approximately 12 hours. Fig. (**2**) illustrates the process of using the diffusion model to generate an example medical image from nonsensical mosaic noise by sampling at different stages.

In the generation experiments, the trained model was capable of producing new chest X-ray images with highly similar features to those in the original dataset, but with obviously different anatomical details. As a visual evaluation, comparisons between the randomly sampled training set and generated results are shown in Fig. (**3A** and **B**), respectively.

### 3.2 Quantitative Evaluation

Fig. (**4**) illustrates the fitting of the generative model to the original dataset during training, in the form of the changes in the loss function. The figure shows the variation of the loss function over 40 training epochs for models trained with L1 loss (blue), L2 loss (orange), and Huber loss (green), respectively.

To obtain a more accurate estimation of the FID value, this study generated 20,480 samples using the trained model. Each generation process took approximately 18 hours. The quanti-tative FID results for different loss functions and feature vector dimensions are presented in Table **1**, indicating optimal parameter settings for model training.

## 4 DISCUSSION

This work utilized MedMNIST v2 as a collection of datasets similar to MNIST. Its advantages for small image processing tasks have been well demonstrated [14], providing more pronounced results at lower computational costs. These characteristics have made it extremely suitable for exploratory pre-experiments where efficiency is of higher priority. Potentially valuable clues and methodological tests for subsequent in-depth research and clinical validation can be prepared. The small size and low resolution of the dataset also enabled rapid testing experiments in this preliminary feasibility study using low-end hardware configurations.

**Fig. (2):**
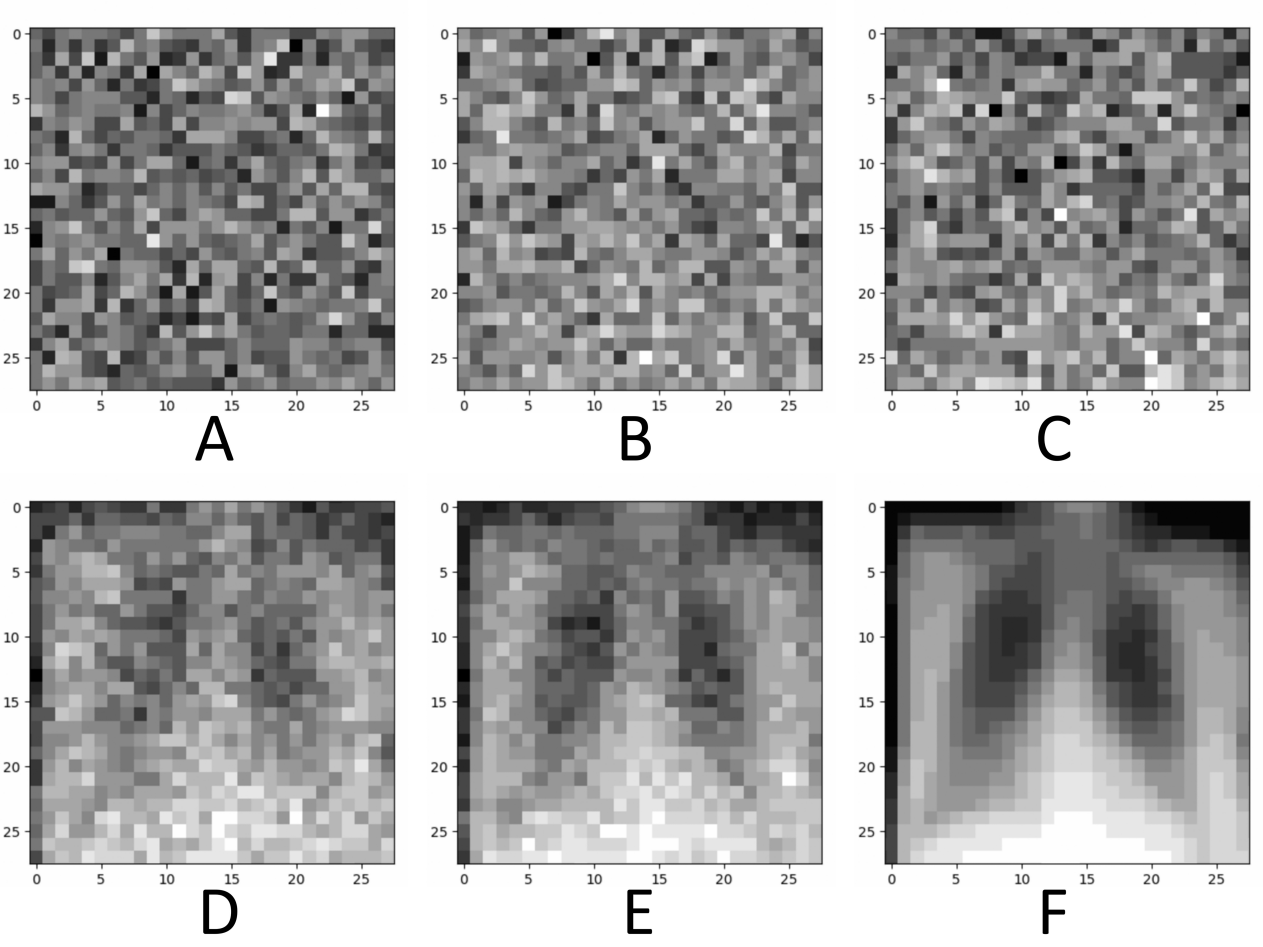
The process of generating an example chest X-ray image using the diffusion model. (**A**), The original Gaussian noise; (**B**), The image after 99 steps of denoising; (**C**), The image after 149 steps of denoising; (**D**), The image after 259 steps of denoising; (**E**), The image after 279 steps of denoising; (**F**), The final generated image after 299 steps of denoising.

**Fig. (3):**
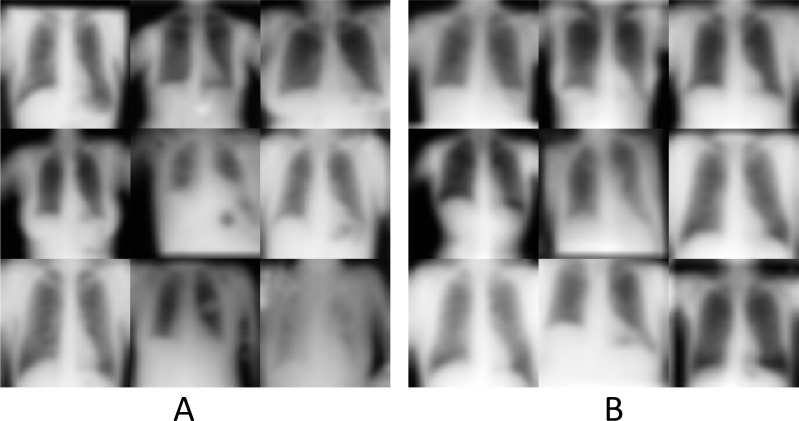
(**A**), Examples of the original images from the ChestMNIST subset in MedMNIST v2; (**B**), Examples of chest X-ray images generated using Huber loss after 40 epochs of training.

As shown in Fig. (**2**), the introduction of noise in diffusion models led to information attenuation (**2A**). The model attempted to predict and reconstruct the original data by progressively denoising the Gaussian noise (Fig. **2B**-**E**). After multiple iterations, the trained diffusion model was capable of learning and generating new images from the given noisy inputs (Fig. **2F**). Visual comparison performed by 3 clinicians suggested that the generated data (Fig. **3B**) maintained high similarity with the original samples (Fig. **3A**) in terms of feature style. However, the generated images exhibited desirable individual variability, such as differences in body shape, posture, lung volume, gray value, field of view, and BMI, *etc*., potentially offering a new image augmentation method for training medical AI models especially in scenarios with limited data availability, such as the increasing legislative restrictions on the acquisition, mining, and sharing of medical data globally [3]. The AI-based data augmentation may also facilitate the rapid development of high-quality models for emergency applications, such as public health events [15].

As a theoretical explanation, the model's outstanding ability to generate medical images with a similar style but different details can be attributed to its inherent randomness and divergence during the sample generation process. These characteristics endowed the generated data with higher degrees of freedom. The latent space information learned by the model during the forward process not only increased the number of generated samples but also enhanced their diversity of details. As a result, diffusion models demonstrated both flexibility and efficiency in generating medical image data.

According to relevant literature on loss functions, the Huber loss displayed more pronounced advantages than L1 and L2 losses in other algorithms, such as deep convolutional dictionary learning [16]. It was also demonstrated that the judicious use of Huber loss can effectively enhance the robustness of diffusion models against data corruption [17]. Compared with traditional loss functions like mean squared error, the Huber loss used in this study exhibited greater stability in handling outliers and remained sensitive to larger errors. Specifically, when the prediction error was lower than a certain threshold, Huber loss behaved similarly to mean squared error, making it more sensitive to small errors. In contrast, for the large prediction errors, Huber loss resembled absolute error, endowing it with stronger robustness against outliers and the ability to produce sparse solutions. As shown in Fig. (**4**), the comparison between the actual and predicted samples in each epoch suggested that the Huber loss function achieved better robustness by balancing the weights of mean squared error (L2 loss) and absolute error (L1 loss). The total value of the loss function converged rapidly and remained stable at a very low level during the whole training process. The trained model also demonstrated good performance in terms of fitting and robustness with respect to the original sample data, echoing the reported effectiveness of the Huber loss function in such tasks.

According to the definition, a lower FID value indicates better similarity between the generated images and the real images. However, there has been no consensus on the golden standard of FID values, which vary depending on the specific application and dataset. Empirically, FID values below 20 were considered indicators of acceptable high-quality generation in the literature [18]. Therefore, all FID values based on different loss functions and feature vector dimensions were considered acceptable, as shown in Table **1**. It was also observed that the FID using a feature vector dimension of 64 was consistently lower than that of 2048. As possible explanations, the lower-dimensional feature vectors may better capture the global features of the data, yet higher dimensions may introduce more details and complexity. As a strategy of parameter optimization during the model training and evaluation based on datasets of various complexities, FID values using different dimensions can be assessed to improve the effectiveness of the model.

Limited by the computing resources, the low resolution (28×28) of MedMNIST v2 used in this feasibility study might restrict the clinical utility of the generated images. In the future, high-resolution datasets (such as 256×256) and more anatomic sites should be used to further validate and improve the proposed method. More comprehensive evaluations based on downstream tasks are also desirable [19], such as automated segmentation and computer-assisted diagnosis. Moreover, manual evaluation is recommended before applying the AI-generated images to clinical model training or testing.

## CONCLUSION

This preliminary study proposed and validated the feasibility of generating medical images with similar styles but different anatomic details using diffusion models, potentially establishing a new data augmentation method for medical AI applications with limited data availability. By examining the dependence on the dimensionality of feature vectors and the complexity of loss functions, optimal training parameters were also proposed for specific scenarios. The proposed method is potentially applicable to improve the efficiency and accuracy of AI-based diagnostic or therapeutic radiology by increasing the volume and diversity of datasets without the time-consuming, expensive, and ethically risky image accumulation in the conventional process.

## Figures and Tables

**Fig. (1):**
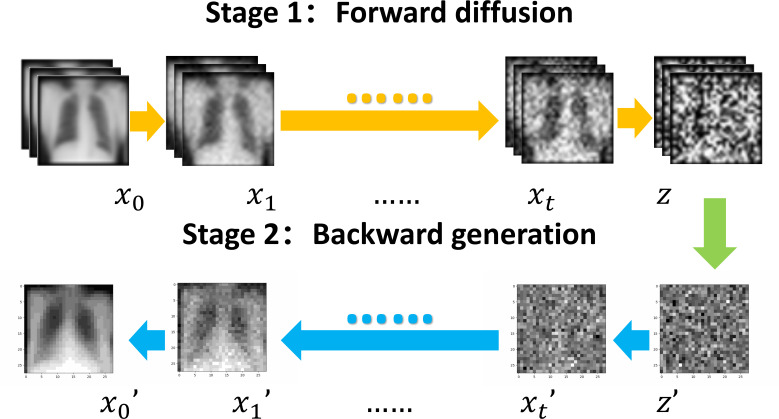
The forward learning and back propagation processes of the diffusion models.

**Fig. (4):**
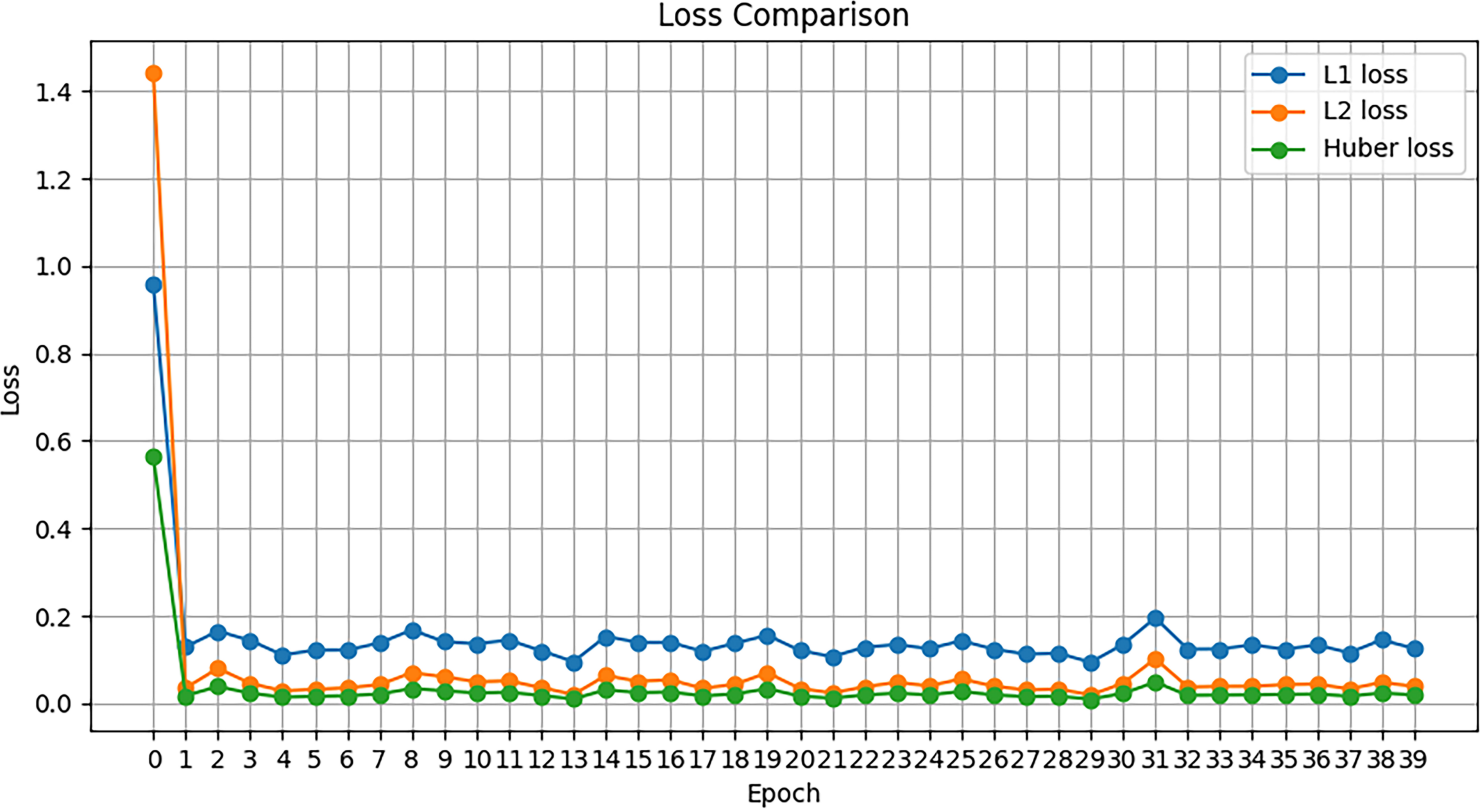
Variation of the loss function with epoch increase during the model training. The x-axis represents the number of epochs for model training, and the y-axis represents the value of the corresponding loss function.

**Table 1:** FID values for different loss functions and feature vector dimensions.

**Loss**	**Dim=2048**	**Dim=64**
L1	16.6	0.99
L2	15.5	0.85
Huber	15.2	0.88

## Data Availability

The dataset used in this study is MedMNIST v2, which is publicly available at the following link: https://github.com/MedMNIST/MedMNIST. Access to the dataset is unrestricted and can be obtained directly through the provided link.

## LIST OF ABBREVIATIONS

AI: Artificial intelligence
BMI: Body Mass Index
FID: Fréchet Inception Distance
GANs: Generative Adversarial Networks
** GPU **: Graphics Processing Unit
** L1 loss **: Mean Absolute Error Loss
** L2 loss **: Mean Squared Error Loss
** ResNet **: Residual Network
VAEs: Variational Autoencoders
